# Ischemia–Reperfusion Injury at Time-Zero Biopsy as a Prognostic Factor in Predicting Liver Graft Outcome in Egyptian Living Donor Liver Transplanted Patients

**DOI:** 10.1155/ijh/9113107

**Published:** 2025-03-06

**Authors:** Aliaa Sabry, Hazem Zakaria, Doha Maher, Randa Mohamed Seddik, Ali Nada

**Affiliations:** ^1^Department of Hepatology & Gastroenterology, Menoufia University, National Liver Institute, Shebin El-Kom, Menoufia, Egypt; ^2^Department of Hepatobiliary Surgery, Menoufia University, National Liver Institute, Shebin El-Kom, Menoufia, Egypt; ^3^Department of Pathology, Menoufia University, National Liver Institute, Shebin El-Kom, Menoufia, Egypt; ^4^Department of Tropical Medicine, Faculty of Medicine, Menoufia University, Shebin El-Kom, Menoufia, Egypt

**Keywords:** graft dysfunction, histological grading, ischemia–reperfusion injury, liver transplantation, survival

## Abstract

**Background and Aims:** Ischemia–reperfusion injury (IRI) is believed to contribute to the early dysfunction of the graft as well as the survival of the patients following liver transplantation (LT). This study is aimed at ascertaining the role of time-zero biopsies in predicting early graft dysfunction and 5-year patient survival after living donor liver transplantation (LDLT).

**Patients and Methods:** From February 2012 to August 2017, time-zero biopsies were obtained from 60 patients. Histological grading of time-zero biopsies was performed to identify the severity of IRI. Patients were divided into two groups: no or minimal to mild IRI versus moderate to severe IRI.

**Results:** Time-zero biopsies of 60 liver allografts revealed no or minimal to mild IRI (*n* = 38, 63.3%) (Group 1) versus moderate to severe IRI (*n* = 22, 36.7%) (Group 2). Group 2 recipients indicated a significant increase in serum bilirubin and a higher incidence of early graft dysfunction. There were significant survival differences between the two groups (*p* = 0.033), and the rate of death was higher in the moderate to severe IRI group. Recipient age, steatosis, and longer CIT were identified as independent predictors of moderate to severe IRI.

**Conclusion:** Time-zero biopsies with moderate to severe IRI upon biopsy can predict adverse clinical outcomes following LT.

## 1. Introduction

Liver transplantation (LT) has gained widespread acceptance as a viable curative therapy option for end-stage liver disease or primary hepatic cancer. Patients' outcomes following LT have improved because of advancements in perioperative management such as surgical technique, perioperative care, and immunosuppressive medication [1–4]. Another important factor in improving outcomes is meticulous graft selection [5]. Hepatic ischemia–reperfusion injury (IRI) is a degenerative pathological process that occurs in some liver transplant recipients. It begins with ischemia-mediated cellular damage and subsequently paradoxically worsens with reperfusion of the liver. This can result in graft loss and an increased risk of early allograft dysfunction (EGD) [6]. Cellular and molecular factors have a significant role in the IRI of the liver. Metabolic disruptions and ATP depletion cause early IRI-induced cell death. IRI is exacerbated by releasing reactive oxygen species (ROS) and inflammatory cytokines from activated neutrophils and macrophages that infiltrate the liver after reperfusion. At last, activation of hepatic stellate cells (HSCs) may cause allograft fibrosis [7, 8]. Tissue damage is accelerated when inflammatory cytokines and chemokines are released, leading to the activation of neutrophils and macrophages [9, 10].

Using animal LT models, extensive research has focused on the mechanics of IRI and strategies to mitigate its negative effects [11–14]. These results suggest that both donor and recipient features and ischemia time may impact the development of hepatic IRI. Several reports on hepatic IRI in human LT have been published, primarily relying on histologic evaluation assessing IRI [15–18]. Liver IRI was shown to be associated with negative LT effects including biliary complications after the operation, acute rejection, and EGD [15–19].

Therefore, we aimed to examine the relationship between the severity of IRI, as determined by the histological grading of time-zero biopsies taken after graft revascularization, and patient and graft outcomes. Moreover, we performed a survival analysis of the recipients over 5 years.

## 2. Patients and Methods

### 2.1. Study Design and Study Participants

**Figure 1:**
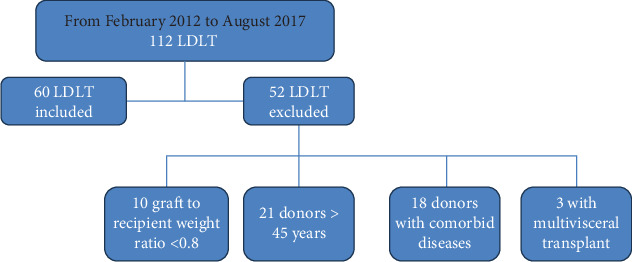
Flowchart of the patient cohort.

This retrospective study of prospectively collected data on liver transplant cases was performed from February 2012 to August 2017, with a follow-up ending in September 2022. Out of all the LTs performed at the National Liver Institute, Menoufia University, 60 grafts were biopsied. Patients who required multivisceral transplants, patients with actual graft-to-recipient weight ratio less than 0.8%, and donors older than 45 years or with chronic diseases (DM, hypertension, and cardiac problems) were excluded from our study (Figure 1). During the preoperative period, all patients received routine triple immunosuppressive medications. All liver grafts were obtained from living donors, entailing both related and unrelated recipients (although most were from related recipients). Data collected comprised pretransplant demographic information (age, gender, indication for LT, comorbidity, laboratory information, and MELD score), zero biopsy histological findings, donor characteristics (age, gender, and laboratory data), warm ischemia time (WIT), cold ischemia time (CIT), and patient/graft outcomes, together with postoperative laboratory data in the first after LT week. The CIT was described as the duration between the donor's perfusion with preservation solution and the liver's retrieval from cold storage. WIT refers to the time elapsed between removing the liver from the ice and reperfusing it with the recipient's blood (using either arterial, portal, or both pathways). Early graft dysfunction was defined by the presence of one or more of the following criteria: a bilirubin level of 10 mg/dL or higher on the seventh postoperative day, an international normalized ratio (INR) of 1.6 or higher on the seventh postoperative day, or aspartate aminotransferase (AST) and alanine aminotransferase (ALT) levels above 2000 IU/L within the initial week. Survival of the patient was determined as the time interval between the transplantation procedure and death due to any cause.

### 2.2. Ethical Approval

This research received the approval of the ethical committee of the National Liver Institute, Menoufia University (IRB: 00560/2024), in compliance with the Helsinki Declaration. After explaining the research questions and objectives to each participant, informed consent was provided before participating.

### 2.3. Assessment of Time-Zero Biopsies and IRI Severity

Intraoperatively, Tru-Cut needle biopsies were taken from the liver roughly 2 or 3 h after portal reperfusion, before the abdomen was surgically closed. Biopsy samples were maintained in paraffin and formalin, and the paraffin blocks of time-zero wedge biopsy were serially sectioned and then stained with hematoxylin and eosin.

Histological examination was done for the following changes: neutrophilic infiltration, hepatocyte apoptosis, steatosis, hemorrhage, hepatocyte ballooning degeneration, and cholestasis. Neutrophilic infiltration is defined as the presence of neutrophils within sinusoids as single cells or clusters and graded as follows: 0: no sinusoidal neutrophils, 1: mild infiltration, 2: moderate infiltration, and 3: severe infiltration [16]. The severity of IRI was determined by polymorphonuclear neutrophil (PMN) infiltration and hepatocellular necrosis. It was then classified into several grades using a scoring system ranging from no injury to minimal, mild, moderate, and severe injury [20].

**Figure 2:**
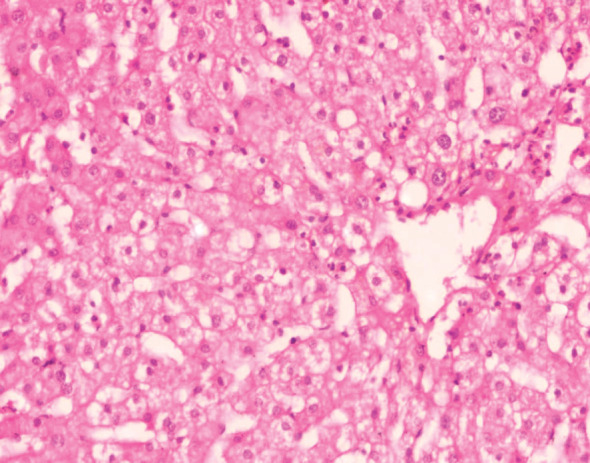
Time-zero liver biopsy showing mild ischemia/reperfusion injury with mild neutrophilic infiltrate with detachment of single hepatocytes from basement membrane (IHC × 200).

**Figure 3:**
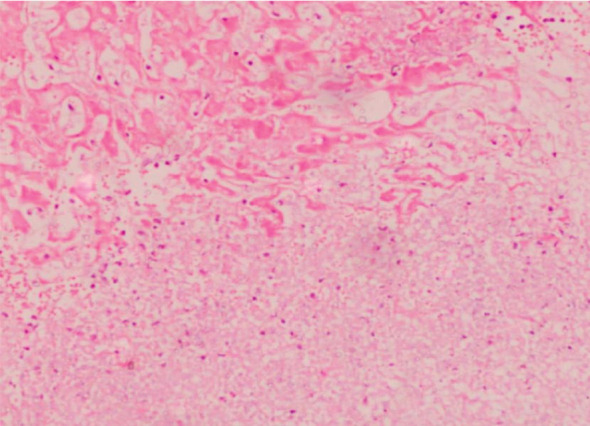
Time-zero liver biopsy showing severe ischemia/reperfusion injury with confluent/zonal coagulative necrosis and hepatocyte loss with neutrophilic infiltrate (IHC × 200).

**Figure 4:**
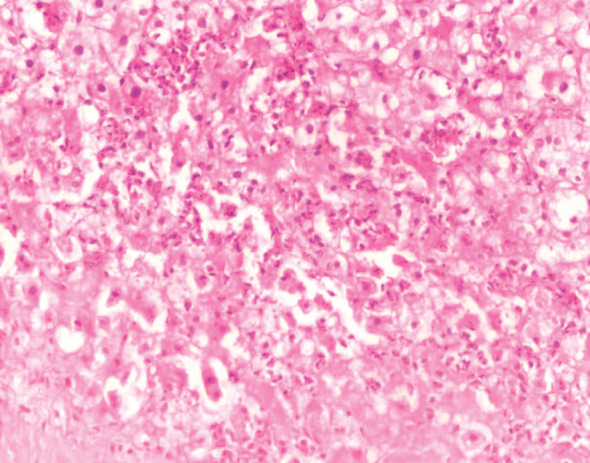
Time-zero liver biopsy showing moderate ischemia/reperfusion injury with presence of clustered (> 5) neutrophils with hepatocyte dropout (IHC × 200).

No IRI severity assessment revealed the absence of inflammatory cells in the normal hepatocytes and sinusoids. The severity of minimal IRI was characterized by a sparse infiltration of individual neutrophils within the sinusoids, without any hepatocyte loss. The mild severity of IRI was identified by a sporadic detachment of individual hepatocytes from the basement membrane or the presence of acidophilic substances, accompanied by a minimal infiltration of neutrophils in the sinusoids, primarily consisting of isolated cells and a few tiny clusters (Figure 2). Severe IRI was identified by the extensive loss of hepatocytes in a zonal or confluent pattern. This damage was typically observed in the perivenular region, while more severe cases could manifest as bridging necrosis or periportal confluent necrosis. An inflammatory infiltration composed of neutrophils was observed in these regions, with additional clusters of neutrophils in more distant sinusoids (Figure 3). Moderate IRI is characterized by neutrophil clusters, with more than five neutrophils present, some of which are accompanied by hepatocyte necrosis or loss (Figure 4). It falls between mild and severe forms of IRI. Crucially, if zonal necrosis was noted without neutrophil infiltration, it was not classified as reperfusion damage but rather as an ischemic lesion in the donor. The 60 grafts were further categorized based on the severity of IRI into two groups: those with no or minimal to mild IRI and those with moderate to severe IRI.

The presence of macrovesicular steatosis was evaluated and the degree of steatosis was measured by estimating the proportion of liver tissue occupied by steatotic vacuoles, using the following method. The distribution of hepatocytes is classified as follows: zero (none), less than 5% of hepatocytes; one (mild), 5%–33% of hepatocytes; two (moderate), 33%–66% of hepatocytes; and three (severe), over 67% of hepatocytes [21].

### 2.4. Statistical Analysis of the Data

The acquired data was collected and analyzed employing the SPSS program (Statistical Package for the Social Science software, version 26) on an IBM-compatible computer. The continuous variables existed as the mean ($\overset{®}{x}$) plus or minus the standard deviation (SD) and the range. To compare the groups, Student's *t*-test (*t*) was used for normally distributed data, whereas the Mann–Whitney *U* test was employed for nonnormally distributed data. Categorical variables were compared using the chi-square test or Fisher's exact test for the two groups. The overall survival analysis was done via Kaplan–Meier statistics, along with the log-rank test to determine the significance. A multivariate regression analysis was conducted to discover variables for IRI. A *p* value less than 0.05 was considered statistically significant.

## 3. Results

### 3.1. Exploratory Data Analysis, Demographic Features, and Clinical Characteristics of Study Participants

A total of 60 liver transplant cases participated in this study. The mean recipient age was 42.17 ± 12.24 years; 86.7% of the patients were male. HCV-related liver disorders were the main reason for LT (*n* = 48, 80.0%). Hepatocellular carcinoma (within Milan criteria) was present in 22 patients (36.7%). As regards comorbid diseases, 10 (16.7%) recipients gave a history of diabetes mellitus and 13 (21.7%) had essential hypertension. The mean body mass index of recipients was 24.33 ± 3.84, and their mean MELD score was 15.77 ± 5.14. Eighteen grafts (30% of total recipients) had steatosis, 18.3% were mild, and 11.7% showed moderate steatosis (Table 1).

### 3.2. Comparison of Participants' Characteristics, Perioperative Data, and Outcomes According to the Severity of IRI

According to IRI severity, recipients were categorized into the following: no IRI: *n* = 9, 15%; minimal: *n* = 6, 10%; mild: *n* = 23, 38.3%; moderate: *n* = 12, 20%; and severe: *n* = 10, 16.7%. The recipients were further categorized into two groups: no or minimal to mild IRI (*n* = 38, 63.3%) versus moderate to severe IRI (*n* = 22, 36.7%). Preliver transplant factors, such as recipient characteristics, perioperative data, and graft outcomes, were contrasted by IRI severity. IRI severity was positively connected with older recipient age as patients with moderate to severe IRI were older than those with no or minimal to mild IRI (*p* = 0.03). Additionally, it was correlated with the degree of hepatic steatosis (6.7% vs. 23.3%, *p* < 0.001) (Table 1).

Regarding operative data, the CIT was longer in the moderate to severe IRI group (455.45 ± 30.27 min vs. 249.34 ± 73.07 min, *p* < 0.001). Also, WIT was significantly longer in the moderate to severe IRI group (51.91 ± 6.75 min vs. 40.39 ± 3.93 min, *p* < 0.001) and the graft-to-recipient weight ratio (GRWR) was higher in no or minimal to mild IRI group (1.43 ± 0.46 vs. 1.19 ± 0.25, *p* = 0.032) (Table 2).

Table 3 displays the outcomes comparing postoperative data after 1 and 7 days by IRI severity. There was no significant difference between the two groups as regards serum bilirubin and AST and ALT levels at postoperative Day 1. When comparing the two groups at postoperative Day 7, the serum bilirubin level exhibited a notable increase in the moderate to severe IRI group (*p* = 0.034). However, there was no significant variance between the two groups considering the other parameters mentioned in this table.

The rate of early graft dysfunction was significantly higher in the moderate to severe IRI group than no or minimal to mild group (eight cases, 36.4%; one case, 2.6%, resp.; *p* < 0.001) (Table 4).

Table 5 indicates a direct significant association between the incidence of early graft dysfunction and the survival rate over 5 years (*p* < 0.001).

**Figure 5:**
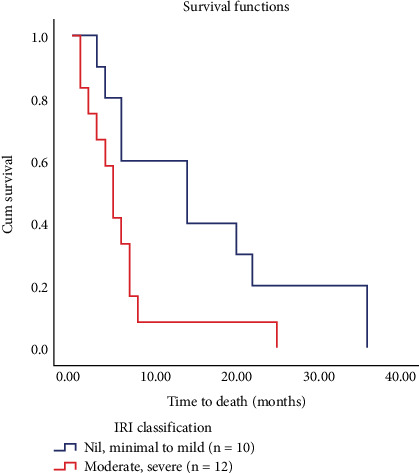
Analysis of overall survival (Kaplan–Meier estimation) in studied patients with IRI.

As regards the survival analysis, there were significant changes among the two groups (*p* = 0.033) and the rate of death was higher in the moderate to severe IRI group. There were 10 deaths in the subgroup nil or minimal to mild IRI and 12 deaths in the subgroup moderate to severe IRI. There were 5 deaths at 3 months, 10 deaths at 1 year, and 7 deaths at 3 years, and there was no mortality recorded in any subgroup at 5 years after transplant (Figure 5 and Table 6).

### 3.3. Multivariate Analysis for Risk Factors of IRI

A multivariate logistic regression analysis was conducted to determine the risk factors associated with moderate to severe IRI. Recipient age (*p* = 0.039, odds ratio (OR): 1.178, and 95% confidence interval (CI): 1.009–1.375), steatosis (*p* = 0.006, OR: 19.096, and 95% CI: 2.313–157.654), and longer CIT (*p* = 0.0014, OR: 1.078, and 95% CI: 1.015–1.144) were recognized as separate indicators of moderate to severe IRI (Table 7).

## 4. Discussion

IRI is a significant and potentially life-threatening consequence that greatly impacts the success of LT. It contributes to up to 10% of early organ failure, resulting in an amplified occurrence of both acute and chronic rejection [22–24]. By analyzing the correlation between the histological grade of IRI severity and patient outcomes, doctors can enhance the selection of grafts and perioperative settings, leading to better post LT patient care and results. Hence, we attempted to establish a connection between the influence of IRI, as assessed by histological grading of biopsies taken immediately after graft revascularization, and the outcomes of the graft, namely, EGD and patient survival 5 years after LT.

In our study, we found that IRI severity was related to recipient age, as the older patients had a higher rate of moderate to severe IRI. In a study performed by Sahmeddini et al., it was reported that a recipient age > 60 years was a strong predictor of postreperfusion syndrome (PRS) [25]. However, in another study, there was no relation between the patient's age and the risk of IRI development [26].

In addition, there was a significant connection between the severity of IRI and the extent of hepatic steatosis (*p* < 0.001). This finding aligned with earlier publications that have documented steatotic grafts as higher risk grafts for hepatic IRI [27]. Moreover, Chung et al. showed that graft steatosis might increase the risk for IRI after LT [28].

It was also found that CIT was longer in the moderate to severe IRI group (*p* < 0.001). A previous study in Chinese LT patients elucidated that prolonged CIT contributed to a higher incidence of IRI, which was associated with adverse outcomes [29]. Additionally, Siniscalchi et al. documented that long-term CIT was the only predictor for IRI [30].

Similarly, WIT was significantly longer in the moderate to severe IRI group than in the no or minimal to mild group (*p* < 0.001). WIT, especially if prolonged, could deteriorate the cellular metabolic deficit because of the toxic metabolites present in venous blood. A study indicated that WIT was a possible risk factor for IRI [31]. Conversely, another study revealed that, in terms of WIT, no statistically significant difference was observed between patients with and without IRI [30].

When comparing the 7-day postoperative liver function tests, serum bilirubin level was significantly higher in the moderate to severe IRI group (*p* = 0.034). Nonetheless, there was no significant variance between the two groups for the other parameters (including transaminases, serum albumin, canalicular enzymes, and INR).

These laboratory profile abnormalities in our trial align with findings of Shahbazi et al. [32] and Mansour et al. [33] who analyzed the degree of correlation between time-zero biopsy findings and early postoperative laboratory data. They testified a significant association between the presence of apoptotic bodies (hepatic necrosis) and liver enzyme elevation in the early posttransplant period. On the other hand, they noted a significant relationship between neutrophilic aggregates and an elevated total bilirubin. Moreover, they concluded that it was a reflection of endothelial cell damage resulting from cold ischemia [32, 33].

In our series, a direct significant correlation between the occurrence of early graft dysfunction and the survival rate over 5 years was confirmed (*p* < 0.001). Moreover, regarding the survival analysis, there was a significant variance between the two groups (*p* = 0.033) and the rate of death was higher in the moderate to severe IRI group.

In addition to our previous findings, it was revealed that patients with moderate IRI grafts exhibited the greatest levels of transaminases and the highest prevalence of EGD. Furthermore, there was a direct relationship between the severity of IRI and the lower survival rate of the graft at 6 months. These findings indicate that the severity of histological IRI is a marker for hepatocellular injury in the short term after LT [24].

In line with our results, Ali et al. reported a link between severe IRI and an increased rate of primary graft nonfunction (PNF), EAD, and re-LT within 90 days. They also displayed that poor 5-year graft survival was only linked with severe IRI [15].

Likewise, the association between the occurrence of IRI and the postoperative poor liver graft and the patient's outcomes was proved. It was found that the incidence of PNF and mortality in the IRI group was significantly higher than in the other group [25].

Several other studies suggested time-zero biopsy IRI grading as a predictor of PNF, acute rejection, EGD, and biliary complications [16–18]. Conversely, other studies did not report any significant association between IRI grade and early graft outcome [33–35].

In our work, multivariate logistic regression analysis for risk factors of IRI identified that older recipient age, presence of steatosis (*p* = 0.006, OR: 19.096, and 95% CI: 2.313–157.654), and longer CIT (*p* = 0.0014, OR: 1.078, and 95% CI: 1.015–1.144) as independent predictors of moderate to severe IRI.

Ito et al. determined that liver steatosis, specifically when it reaches 20% or more of large droplet macrovesicular steatosis, is independently related to the development of EGD in IRI grafts [24]. Nevertheless, the multivariate analysis did not establish steatosis as a causative factor for IRI itself. Furthermore, there were no variations in graft survival based on the severity of IRI in grafts with significant steatosis. The authors hypothesized that the limited quantity of steatotic grafts in their study would have underestimated the influence of steatosis on IRI [24].

Unlike the previous study, prolonged CIT was identified as an additive risk for severe hepatic IRI in steatotic livers [36]. Moreover, Ito et al. reported that hepatic IRI could be considered reversible in cases that performed grafts with no steatosis and with shorter CIT [24].

This study has some limitations due to its single-center retrospective analysis and small sample size of 60 patients. Thus, further research is required to validate our results.

## 5. Conclusion

IRI is an inevitable process during LT that can affect both graft function and patient survival. Time-zero biopsies with moderate to severe IRI upon biopsy can predict adverse clinical outcomes after LT.

## Figures and Tables

**Table 1:** The characteristics of studied participants.

**Variable**	**IRI**							
	**Nil or minimal to mild (** **n** = 38**)**		**Moderate to severe (** **n** = 22**)**		**Total (** **n** = 60**)**		**Test**	**p** ** value**
	**No.**	**%**	**No.**	**%**	**No.**	**%**		
Recipients								
Sex								
Male	33	86.8	19	86.4	52	86.7	FE = 0.003	1.000
Female	5	13.2	3	13.6	8	13.3		
Age (years)								
Mean ± SD	39.61 ± 13.83		46.59 ± 7.14		42.17 ± 12.24		*U* = 2.169	**0.030**⁣^∗^
Range	3–53		27–56		3–56			
Indication for LT								
HCV	30	78.9	18	81.8	48	80	*χ* ^2^ = 5.631	0.584
HCC	14	36.8	8	36.4	22	36.7		
PBC	1	2.6	3	13.6	4	6.7		
PSC	1	2.6	1	4.5	2	3.3		
Biliary atresia	2	5.3	0	0.0	2	3.3		
HBV	1	2.6	1	4.5	2	3.3		
Liver storage disease	2	5.3	0	0.0	2	3.3		
Diabetes mellitus								
Present	8	21.1	2	9.1	10	16.7	FE = 1.435	0.299
Absent	30	78.9	20	90.9	50	83.3		
Hypertension								
Present	8	21.1	5	22.7	13	21.7	FE = 0.023	1.000
Absent	30	78.9	17	77.3	47	78.3		
BMI (kg/m^2^)								
Mean ± SD	24.00 ± 3.41		24.91 ± 4.51		24.33 ± 3.84		*t* = 0.819	0.418
Range	20–33		20–33		20–33			
MELD								
Mean ± SD	15.97 ± 5.28		15.41 ± 4.98		15.77 ± 5.14		*t* = 0.414	0.681
Range	7–34		8–34		7–34			
Steatosis in the graft								
Nil	34	89.5	8	36.4	42	70.0	*χ* ^2^ = 21.151	<0.001^∗^
Mild	4	10.5	7	31.8	11	18.3		
Moderate	0	0.0	7	31.8	7	11.7		

*Note:* Bold means *p* value is significant. *χ*^2^, chi-squared test; *U*, Mann–Whitney test.

Abbreviations: BMI, body mass index; FE, Fisher's exact test; HBV, hepatitis B virus; HCC, hepatocellular carcinoma; HCV, hepatitis C virus; IRI, ischemia–reperfusion injury; MC, Monte Carlo correlation; MELD, model of end-stage liver disease; PBC, primary biliary cholangitis; PSC, primary sclerosing cholangitis; SD, standard deviation; *t*, Student's *t*-test.

⁣^∗^Significant *p* value.

**Table 2:** Operative data of studied participants.

**Variable**	**IRI**			
	**No or minimal to mild (** **n** = 38**)**	**Moderate to severe (** **n** = 22**)**	**Test of sig.**	**p** ** value**
Operative time (hours)				
Mean ± SD	11.20 ± 2.37	11.52 ± 2.33	*t* = 0.515	0.609
Range	7–15	7–15		
Blood units				
Mean ± SD	2.89 ± 2.85	2.73 ± 2.82	*U* = 0.351	0.726
Range	0–11	0–10		
CIT (minutes)				
Mean ± SD	249.34 ± 73.07	455.45 ± 30.27	*t* = 12.566	**< 0.001**⁣^∗^
Range	130–455	375–495		
WIT (minutes)				
Mean ± SD	40.39 ± 3.93	51.91 ± 6.75	*t* = 8.371	**< 0.001**⁣^∗^
Range	35–56	35–65		
Anhepatic (hours)				
Mean ± SD	3.37 ± 0.77	3.10 ± 0.57	*t* = 1.427	0.159
Range	2–5	2–4		
GFR				
Mean ± SD	87.89 ± 14.55	83.09 ± 14.63	*t* = 1.228	0.226
Range	67–114	62–110		
GRWR				
Mean ± SD	1.43 ± 0.46	1.19 ± 0.25	*t* = 2.201	**0.032**⁣^∗^
Range	0.89–2.5	0.89–1.8		
Weight of native liver (gm)				
Mean ± SD	1389.47 ± 278.06	1478.18 ± 341.00	*t* = 1.037	0.307
Range	1050–2200	1050–2200		
Weight of graft (gm)				
Mean ± SD	834.21 ± 86.29	881.82 ± 111.85	*t* = 1.845	0.070
Range	700–1050	700–1050		

*Note:* Bold means *p* value is significant. *U*, Mann–Whitney test.

Abbreviations: CIT, cold ischemia time; GFR, glomerular filtration rate; GRWR, graft-to-recipient weight ratio; *t*, Student's t-test; WIT, warm ischemia time.

⁣^∗^Significant *p* value.

**Table 3:** Postoperative data after 1 day and 1 week in studied participants.

**Data**	**IRI**			
	**No or minimal to mild (** **n** = 38**)**	**Moderate to severe (** **n** = 22**)**	**Test of sig.**	**p** ** value**
POD 1 data				
Total serum bilirubin				
Mean ± SD	6.46 ± 3.08	6.55 ± 2.09	*U* = 0.277	0.782
Range	1.7–13	2.4–10		
AST				
Mean ± SD	176.08 ± 158.84	118.50 ± 107.99	*U* = 1.613	0.107
Range	18–679	18–495		
ALT				
Mean ± SD	152.68 ± 133.62	104.09 ± 101.53	*U* = 1.382	0.167
Range	24–442	30–442		
POD 7 data				
Total serum bilirubin (mg/dL)				
Mean ± SD	6.09 ± 4.01	8.52 ± 4.55	*U* = 2.121	**0.034**⁣^∗^
Range	0.4–13.6	0.4–13.6		
AST (IU/L)				
Mean ± SD	99.68 ± 93.55	96.59 ± 80.33	*U* = 0.108	0.914
Range	13–305	11.5–300		
ALT (IU/L)				
Mean ± SD	135.79 ± 150.57	94.91 ± 92.47	*U* = 0.999	0.318
Range	5–547	5–400		
Serum albumin (g/dL)				
Mean ± SD	2.99 ± 0.37	3.06 ± 0.52	*t* = 0.597	0.553
Range	2.4–4	2.4–4		
Alkaline phosphatase (U/L)				
Mean ± SD	195.00 ± 134.85	189.00 ± 104.74	*U* = 0.015	0.988
Range	34–500	85–480		
GGT (IU/L)				
Mean ± SD	270.24 ± 169.47	227.68 ± 136.99	*U* = 0.706	0.480
Range	34–565	67–565		
Serum creatinine (mg/L)				
Mean ± SD	0.79 ± 0.39	0.81 ± 0.27	*U* = 0.262	0.793
Range	0.2–1.3	0.3–1.2		
INR				
Mean ± SD	1.41 ± 0.29	1.44 ± 0.26	*t* = 0.431	0.668
Range	0.9–1.89	0.9–1.8		
Hemoglobin (g/dL)				
Mean ± SD	8.88 ± 1.01	9.16 ± 0.58	*t* = 1.143	0.285
Range	7–11.5	8.5–10.8		
WBCs (10^3^/L)				
Mean ± SD	8.54 ± 2.77	8.80 ± 2.97	*t* = 0.332	0.741
Range	3.5–12.3	4–12.3		
Platelets (10^9^/L)				
Mean ± SD	99.08 ± 44.24	106.50 ± 51.88	*U* = 0.553	0.580
Range	49–285	56–285		
FK dose				
Mean ± SD	1.36 ± 0.45	1.60 ± 0.83	*U* = 0.358	0.721
Range	0.75–3	0.75–3		
FK level (ng/mL)				
Mean ± SD	8.51 ± 2.07	8.03 ± 1.13	*U* = 0.085	0.932
Range	5.3–10.7	5.3–11		

*Note:* Bold means *p* value is significant. FK, oral tacrolimus; *U*, Mann–Whitney test.

Abbreviations: POD 1, Postoperative Day 1; POD 7, Postoperative Day 7; *t*, Student's t-test.

⁣^∗^Significant *p* value.

**Table 4:** The relation between graft dysfunction and ischemia–reperfusion injury of studied participants.

**Early graft dysfunction**	**IRI**							
	**No or minimal to mild (** **n** = 38**)**		**Moderate to severe (** **n** = 22**)**		**Total (** **n** = 60**)**		**Test**	**p** ** value**
	**No.**	**%**	**No.**	**%**	**No.**	**%**		
Yes	1	2.6	8	36.4	9	15.0	FE = 12.435	**0.001**⁣^∗^
No	37	97.4	14	63.6	51	85.0		

*Note:* Bold means *p* value is significant.

Abbreviation: FE, Fisher's exact test.

⁣^∗^Significant *p* value.

**Table 5:** The relation between graft dysfunction and survival of studied participants.

**Early graft dysfunction**	**Survived (** **n** = 37**)**		**Dead (** **n** = 23**)**		**Total (** **n** = 60**)**		**Test**	**p** ** value**
	**No.**	**%**	**No.**	**%**	**No.**	**%**		
Yes	1	2.7	8	34.8	9	15.0	FE = 11.448	**0.001**⁣^∗^
No	36	97.3	15	65.2	51	85.0		

*Note:* Bold means *p* value is significant.

Abbreviation: FE, Fisher's exact test.

⁣^∗^Significant *p* value.

**Table 6:** An overall survival analysis in studied patients with their IRI.

**Group**	**No. of cases**	**Mean**				**Median**				**Log-rank test**	**p** ** value**
		**Estimate**	**SE**	**95% CI**		**Estimate**	**SE**	**95% CI**			
				**Lower**	**Upper**			**Lower**	**Upper**		
Nil, minimal to mild	10	16.100	3.906	8.445	23.755	14.000	6.197	1.854	26.146	4.537	**0.033**⁣^∗^
Moderate, severe	13	6.167	1.842	2.557	9.776	5.000	0.854	3.326	6.674		
Overall	23	10.682	2.260	6.253	15.111	4.000	0.934	4.169	7.831		

*Note:* Bold means *p* value is significant.

⁣^∗^Significant *p* value.

**Table 7:** A multivariate analysis for predictors of moderate to severe ischemia–reperfusion injury.

**Variable**	**p** ** value**	**Odds ratio**	**95% confidence interval**	
			**Lower**	**Upper**
Recipient age (years)	**0.039**⁣^∗^	1.178	1.009	1.375
Steatosis Present (ref.) Absent	**0.006**⁣^∗^	19.096	2.313	157.654
CIT (minutes)	**0.014**⁣^∗^	1.078	1.015	1.144
WIT (minutes)	**0.265**	0.868	0.676	1.144

*Note:* Bold means *p* value is significant.

Abbreviations: CIT, cold ischemia time; WIT, warm ischemia time.

⁣^∗^Significant *p* value.

## Data Availability

The entirety of the data produced or examined within this investigation is encompassed within this published publication.

## Supporting Information

Supporting Information 1 File S1: This is the protocol submitted to the ethical committee at the National Liver Institute, Menoufia University, Egypt.

Supporting Information 2 File S2: This is an Excel sheet of the data of our work.
