# Supplementary material for: Ischemia–Reperfusion Injury at Time-Zero Biopsy as a Prognostic Factor in Predicting Liver Graft Outcome in Egyptian Living Donor Liver Transplanted Patients
Source: Int J Hepatol. 2025 Mar 6;2025:9113107. doi: 10.1155/ijh/9113107 (PMC11991779; doi:10.1155/ijh/9113107)
Supplement: Supporting Information 1 — File S1: This is the protocol submitted to the ethical committee at the National Liver Institute, Menoufia University, Egypt. [file 9113107.f1.docx]

**Ischemia reperfusion injury at zero time biopsy as prognostic factor in prediction of liver graft outcome in Egyptian Living donor liver transplanted patients**

Ali Nada (1), hazem zakaria(2).Doha maher (3), Randa sedek (4), Aliaa sabry (1)

1 National liver institute Menofia university Department of hepatology gastroenterology .2 National liver institute Menofia university Department of hepatobiliary surgery .3 National liver institute Menofia university Department pathology. 4 Faculty of Medicine Menofia university Department of tropical medicine

**Introduction**

Liver transplantation is an established and successful treatment option for patients with end-stage hepatic failure, with 5-year survival rates in excess of 70%.1,2 Despite this success, early complications persist.2 Although primary nonfunction (PNF) is immediately evident, approximately another 15% of liver transplant recipients suffer progressive graft dysfunction which culminates in either death or the need for retransplantation within the first year.3-5 With the emphasis on expanding the donor pool through the use of marginal or extended criteria donors, being able to predict those patients who will suffer early graft failure after poor initial graft function is becoming increasingly important 6,7 because this identification may enable such patients to undergo retransplantation earlier when they are still relatively well.8

**Aim of the work**

Validity of Ischemia reperfusion injury as prognostic factor in prediction of liver allograft outcome in Egyptian liver transplanted patients

**Patients and Methods**

**Patient Recruitment**

All patients undergoing Living donor liver transplantation at the National liver institute Hospitals’ Transplantation Unit over a 5 year period from between February 2012 to August 2017, time zero biopsy was taken in 60 patients were included, with follow-up

**Assessment of Time-Zero Biopsies**

Protocol Tru-Cut needle biopsies were obtained intraoperatively after complete revascularization of the allograft, typically after a hemostatic pause before the commencement of the biliary anastomosis (approximately 45 minutes to 1 hour) , severity of Ischemia reperfusion injury assessment , severity of steatosis assessment

**Outcomes**

Primary non function graf (PNF) was defined as poor graft function necessitating retransplantation or culminating in death within 14 days; rejection and vascular thrombosis were excluded. Early graft dysfunction was characterized by the presence of 1 or more of the following: serum bilirubin exceeding 9.5 mg/dl on day 7 after operation, international normalized ratio at least 1.6 on day 7, and alanine aminotransferase (ALT) greater than 2000 U/mL within the first 7 days after surgery. survival was defined as the time from transplantation to death from any cause.

**Factors associated with severity of IRI**

All data will be collected and analyzed according to severity of IRI including age of donor and recipients , BMI , causative agent for transplantation ,MELD score , liver function test , renal function test , complete blood count , immunosuppression

**Statistics**

All data will be statistically analyzed

**Refeances**

1- Ahmed A, Keeffe EB. Current indications and contraindications for liver transplantation. Clin Liver Dis 2007;11: 227-247.

2. Keeffe EB. Liver transplantation: current status and novel approaches to liver replacement. Gastroenterology 2001;120:749-762.

3. Olthoff KM, Kulik L, Samstein B, Kaminski M, Abecassis M, Emond J, et al. Validation of a current definition of early allograft dysfunction in liver transplant recipients and analysis of risk factors. Liver Transpl 2010;16:943- 949.

4. Waki K. UNOS liver registry: ten year survivals. Clin Transpl 2006:29-39.

5. Taniguchi M. Liver transplantation in the MELD era— analysis of the OPTN/UNOS registry. Clin Transpl 2012: 41-65.

6. Mallik M, Callaghan CJ, Hope M, Gibbs P, Davies S, Gimson AE, et al. Comparison of liver transplantation outcomes from adult split liver and circulatory death donors. Br J Surg 2012;99:839-847.

7. Mullhaupt B, Dimitroulis D, Gerlach JT, Clavien PA. Hot topics in liver transplantation: organ allocation— extended criteria donor—living donor liver transplantation. J Hepatol 2008;48(suppl 1):S58-S67.

8. Feng S, Goodrich NP, Bragg-Gresham JL, Dykstra DM, Punch JD, DebRoy MA, et al. Characteristics associated with liver graft failure: the concept of a donor risk index. Am J Transplant 2006;6:783-790.
